# Analyzing Gene Expression from Marine Microbial Communities using Environmental Transcriptomics

**DOI:** 10.3791/1086

**Published:** 2009-02-18

**Authors:** Rachel S. Poretsky, Scott Gifford, Johanna Rinta-Kanto, Maria Vila-Costa, Mary Ann Moran

**Affiliations:** Department of Marine Sciences, University of Georgia (UGA)

## Abstract

Analogous to metagenomics, environmental transcriptomics (metatranscriptomics) retrieves and sequences environmental mRNAs from a microbial assemblage without prior knowledge of what genes the community might be expressing. Thus it provides the most unbiased perspective on community gene expression *in situ*. Environmental transcriptomics protocols are technically difficult since prokaryotic mRNAs generally lack the poly(A) tails that make isolation of eukaryotic messages relatively straightforward ^1^ and because of the relatively short half lives of mRNAs ^2^. In addition, mRNAs are much less abundant than rRNAs in total RNA extracts, thus an rRNA background often overwhelms mRNA signals. However, techniques for overcoming some of these difficulties have recently been developed. A procedure for analyzing environmental transcriptomes by creating clone libraries using random primers to reverse-transcribe and amplify environmental mRNAs was recently described was successful in two different natural environments, but results were biased by selection of the random primers used to initiate cDNA synthesis ^3^. Advances in linear amplification of mRNA obviate the need for random primers in the amplification step and make it possible to use less starting material decreasing the collection and processing time of samples and thereby minimizing RNA degradation ^4^. *In vitro* transcription methods for amplifying mRNA involve polyadenylating the mRNA and incorporating a T7 promoter onto the 3  end of the transcript. Amplified RNA (aRNA) can then be converted to double stranded cDNA using random hexamers and directly sequenced by pyrosequencing ^5^. A first use of this method at Station ALOHA demonstrated its utility for characterizing microbial community gene expression ^6^.

**Figure:**
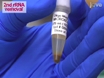


## Protocol

### Working with RNA

Because RNases are ubiquitous and mRNAs degrade rapidly, standard precautions for working in a ribonuclease-free environment must be followed and samples should be processed or preserved as soon as possible following collection.

**Part 1: Environmental RNA Collection** (designed to collect biomass in the 0.2- 3.0 µm size fraction)

Supplies needed: Masterflex tubing Peristaltic Pump 3 µm high volume pleated capsule filter  0.22 µm Supor or polycarbonate 142 mm filter 142 mm filter tower (Geotech Environmental Equipment) 10-20 L Carboy (graduated) Whirl Packs Liquid nitrogen RLT buffer (Qiagen) Beta-mercaptoethanol  Setup: Clean gloves should be worn whenever handling the filters or touching any of the interior components of the filter line. Forceps should be kept in clean, 50 ml conical tubes, preferably filled with ethanol.  To prevent major changes in the transcript pool, keep filtering and sample handling times as short as possible. Place one end of the tubing into the water at the desired depth for sampling. On the opposite end, attach the high volume 3 µm filter. Connect the 3 µm filter to the 0.22 µm filter tower. Direct the outflow from the 0.22 μm into a graduated carboy.

#### Procedure:

1. Run several liters of water through the system (no 0.22 µm filter) to rinse. When finished, remove the tubing from the water while the pump is running to purge the remaining water from the line.
2. Using forceps, place a 0.22 µm filter on the filter tower and close.
3. Place the end of the line back into the water and turn on the pump. Purge the air from both the filters. Monitor the volume of water filtered with the graduation on the carboy.
4. When the desired volume has been filtered, remove the line from the water and purge the remaining water from the system.
5. As soon as the filter is dry, quickly remove the top filter plate and fold the filter. Place the filter into a whirl pack or 15 ml collection tube containing 2 ml of RLT buffer with beta-mercaptoethanol. Snap freeze in liquid nitrogen.

#### Part 2: Total RNA extraction from 0.22 μm filter

*This part uses the QIAGEN RNeasy mini kit with modifications to the manufacturer’s instructions*

1. Prepare bead beating tubes by adding 8 ml of RLT buffer (beta-mercaptoethanol added) to a 50 ml conical tube. Add 1-2 ml of RNA beads from a MoBio Powersoil extraction kit
2. Quickly remove the frozen filter from the freezer and, keeping it in the whirl pack, shatter it with a mallet.  Alternatively, cut the filters in small pieces in the collection tube (15ml) using a sterile razor blade and add it all to the prepared tube (50ml).
3. Add the shattered filter to the prepared tube (50ml), and seal the tube lid with parafilm or lab tape.
4. Vortex for 10 minutes at top speed using a vortexer with an attachment for 50 mL tubes.
5. Centrifuge the 50 ml tube at 5000 rpm for 1 min at room temperature.
6. Remove as much as possible of the lysate and transfer it to a 15 ml tube.
7. Centrifuge the 15 ml tube for 5 min at 5000 x g.
8. Transfer supernatant (~7 ml) to a new 50 ml tube.
9. Add 1 volume (~7 ml) of 100% EtOH to the lysate.
10. Using a 30 ml syringe that fits into the 50 ml tube, draw the lysate up through an 18-21 gauge needle and pass it back out ~5 times. When finished, leave the lysate in the syringe.
11. Continue the extraction with the QIAGEN RNeasy Mini kit. It is helpful to use a vacuum manifold here, since the lysate volume (~ 14ml) is much higher than the original kit protocol (0.7 ml).  Alternatively, the lysate can be drawn through by adding 700 µl to the column, centrifuging, and repeating until all of the lysate has been applied to the column.
12. Place a spin column on the vacuum manifold and apply 700 µl of the sample. Apply vacuum pressure to the manifold to draw down the lysate. Continue adding the lysate from the syringe until all of it has been applied to the column.
13. Add 700 µl buffer RW1 to the column and draw down with vacuum pressure.
14. Add 500 µl buffer RPE to the column and draw down with vacuum pressure.
15. Add a second aliquot of 500 µl buffer RPE to the column and draw down with vacuum pressure.
16. Once the wash has been completely drawn through, remove the column and place in a collection tube.
17. Centrifuge the tube for 1 min at 8000 x g. Discard flow-through.
18. Centrifuge an additional 2 min at 8000 x g to get rid of the EtOH.
19. Transfer column to a new 2 ml collection tube. Pipette 35 µl of RNase-free water directly onto the membrane. Close tube and let stand for 1 min.
20. Centrifuge for 2 min at 8000 x g.
21. Quantify the total RNA spectrophotometrically.

#### Part 3. DNAse treatment

*This part uses the Ambion TURBO DNA-free kit according to the manufacturer’s instructions*

1. Add 3.4 µl 10x DNase I Buffer and 1µl rDNase I to the RNA sample.
2. Incubate at 37ºC for 30min.
3. Vortex the DNase Inactivation Reagent and add 3.4 µl of the inactivation reagent to the sample.
4. Incubate 2 min at room temperature with occasional mixing.
5. Spin at 10.000 x g for 1.5 min at room temperature, then transfer the supernatant to a new tube. Avoid introducing the inactivation reagent into the fresh tube.

#### Part 4. First rRNA removal

*This part uses the Epicentre mRNA-ONLY kit according to the manufacturer’s instructions*

1. Gently mix and briefly centrifuge the mRNA-ONLY 10X Reaction Buffer prior to use.
2. In a sterile (RNase-free) 0.5 ml tube, combine the following reaction components on ice:

**Table:** 

mRNA-ONLY 10X Reaction Buffer	2.0 µl
RNase Inhibitor	0.5 µl
**Total RNA Sample (200 ng-10 µg)**	**16.5 µl**
Terminator Exonuclease (1 Unit)	1.0 µl
**Total volume**	**20.0 µl**
3. Incubate the reaction at 30°C for 60 min in a thermocycler (with heated lid) or water bath.
4. Terminate the reaction with Phenol/EtOH precipitation:
  1. Add RNase-free H2O to a total volume of 200 µl (= 180 µl).
  2. Extract: Phenol:Chloroform (1:1), 1 volume (= 200 µl). Vortex vigorously and then spin 2-5 min at top speed.
  3. Remove aqueous phase (~200 µl).
  4. Add 0.1 volumes of 3 M Sodium Acetate (20 µl) + 2.5 volume of 100% ice cold EtOH (500 µl)
  5. Incubate at -80ºC for 15-30 min.
  6. Pellet at 4ºC, top speed, 30 min; note the position of the tube for later aspiration of sample (pellet can be difficult to see).
  7. Discard supernatant with aspirator or pipette.
  8. Wash with 500 µl 70% ice cold EtOH. Resuspend by vortexing.
  9. Centrifuge for 10 min at top speed. Discard supernatant.
  10. Centrifuge remaining liquid in tube again 10 min at top speed.
  11. Aspirate liquid carefully and dry pellet completely by leaving it open for ~10 min under the hood.
  12. Resuspend in 15-20 µl RNase-free H2O (maximum input volume of MICROBExpress is 15 µl); let it stand for 5 min at room temperature.
  13. Use Bioanalyzer or Experion to check for rRNA contamination and quality of mRNA at this step.
  14. This is a potential stopping point. RNA can be stored at -80ºC storage.

#### Part 5. Second rRNA removal

*This part uses the Ambion MICROBEnrich and MICROBExpress kits according to the manufacturer’s instructions. Both kits can be used multiple times to increase the efficiency of eukaryotic (MICROBEnrich) and prokaryotic (MICROBExpress) rRNA removal. The input of total RNA should be between 2-10 µg. The volume of RNA should be less than 15 µl. Thus, the ideal input is >150 ng/µl. -*

MICROBEnrich

1. Pipette 300 µl Binding buffer into a 1.5ml tube
2. Add 5–100 µg total RNA in a maximum volume of 30µl and vortex gently
3. Add 2 µl of Capture Oligo Mix for 5 µg RNA in Binding buffer. Tap tube gently and briefly spin down the mixture.
4. Denature mixture at 70°C for 10 min
5. Anneal the mixture at 37°C for 1 hr. Prepare the beads during this incubation.
6. Prepare Oligo MagBeads (usually 25 µl) by washing once in nuclease-free water and once in Binding buffer, capturing the beads on a magnetic stand between washes. Store the beads on ice. Warm them up to room temperature 5 min before use.
7. Heat the Wash solution to 37°C in heat block.
8. Add the RNA/capture Oligo Mix to the washed Oligo MagBeads. Very gently mix the tube and spin briefly.
9. Incubate the mix at 37°C for 15 min.
10. Place the tube in the magnetic stand and wait for ~3 min.
11. Aspirate supernatant (contains mRNA) and transfer it to a collection tube on ice.
12. Add 100 µl of pre-warmed Wash solution to captured beads. Gently vortex the beads briefly.
13. Capture beads and recover supernatant. Pool this supernatant with mRNA in the collection tube on ice (~450 µl end volume).
14. Place the collection tube on ice. If a second round is going to be performed, go back to 5.3) section and repeat the procedure. Alternatively, switch immediately to the MICROBExpress kit (5.18, see below).
15. Used OlioBeads can be used a second time. Regenerate the Oligobeads by incubating them with 2 volumes (usually 50 µl) of Regeneration Solution 1 for 1 hr. Capture the beads in a magnetic stand. Wash them twice with 2 volumes of Regeneration Solution 2 (usually 50 µl) and resuspend them in their original volume of Resuspension solution (usually 25 µl). MICROBExpress
16. Pipette 200 µl Binding buffer into a 1.5ml tube.
17. Add 2–10 µg total RNA in a maximum volume of 15µl and vortex gently.
18. Add 4 µl of Capture Oligo Mix to the RNA in Binding buffer. Tap tube gently and briefly spin down the mixture.
19. Denature mixture at 70°C for 10 min.
20. Anneal mixture at 37°C for 15 min Prepare beads during this incubation.
21. Prepare Oligo MagBeads by washing once in nuclease-free water followed by two washes in 50 μl Binding buffer, capturing the beads on a magnetic stand between washes. Resuspend beads in 50 μl Binding buffer and incubate at 37°C until use.
22. Heat the Wash solution to 37°C in heat block
23. Vortex washed Oligo MagBeads gently. Add 50 µl Oligo MagBeads to RNA/capture Oligo Mix. Very gently mix the tube and spin briefly.
24. Incubate mix at 37°C for 15 min.
25. Place the tube in the magnetic stand and wait for ~3 min.
26. Aspirate supernatant (contains mRNA) and transfer it to a collection tube on ice.
27. Add 100 µl of pre-warmed Wash solution to captured beads. Gently vortex the beads briefly.
28. Capture beads and recover supernatant. Pool this supernatant with mRNA in the collection tube on ice (~350 µl end volume).
29. If a second round is going to be performed, go back to section 5.18) and repeat the procedure. Alternatively, proceed immediately to mRNA precipitation.
30. Precipitate and resuspend mRNA:
  1. To the pooled mRNA add 35 µl Sodium Acetate and add 7 µl Glycogen.
  2. Add 1175 µl ice cold 100% EtOH and vortex thoroughly.
  3. Precipitate at -20°C for at least 1h.
  4. Centrifuge for 30 min at 10.000 x g.
  5. Carefully decant and discard the supernatant.
  6. Add 750 µl ice cold 70% EtOH and vortex briefly.
  7. Centrifuge for 5 min at 10.000 x g and discard supernatant.
  8. Do a second 70% EtOH wash.
  9. Briefly respin the tube after discarding the second 70% EtOH wash.
  10. Carefully remove any supernatant with a pipette.
  11. Air dry the pellet for 5 min.
  12. Resuspend the RNA pellet in 25 µl TE buffer or RNase free water
  13. Rehydrate RNA for 15 min at room temperature.
  14. Vortex the sample vigorously if necessary.
31. If it is necessary remove remaining beads, place tube on magnetic stand for ~ 3min and move enriched mRNA to new RNase free tube.
32. Use Bioanalyzer or Experion to check for rRNA contamination and quality of mRNA at this step.
33. This is a potential stopping point. RNA can be stored at -80ºC storage.

#### Part 6: mRNA Amplification

*This part uses the Ambion MessageAmp II- Bacteria  kit according to the manufacturer’s instructions. Calculate master mixes online at www.ambion.com/tools/ma2bact.*

1. Polyadenylation of template RNA:
  1. Place up to 1000 ng of Total RNA (typically 100–500 ng) or up to 500 ng mRNA (typically 10 ng–200 ng) into a sterile RNase-free microfuge tube. Use 6.5 µl of sample and no RNase free water in the first buffer.
  2. Incubate 10 min at 70°C, preferably in a thermocycler.
  3. Remove the RNA samples from the 70°C incubator and centrifuge briefly (~5 s) to collect sample at the bottom of the tube. Place the mixture on ice for 3 min.
  4. Prepare Polyadenylation Master Mix in a nuclease-free tube at room temp in the order shown on the calculation sheet. Assemble enough master mix for all the samples in the experiment, including ≤5% overage to cover pipetting error. Gently vortex to make a homogenous mixture without inactivating the enzyme, then centrifuge for ~5 s to collect the master mix at the bottom of the tube.
  5. Transfer 3.5 µl of Polyadenylation Master Mix to each RNA sample, mix thoroughly by gentle vortexing and follow with a quick spin to collect the reaction.
  6. Place the samples in a 37°C incubator. Incubate reactions for 15 min at 37°C, then centrifuge briefly to collect the reaction at the bottom of the tube.
  7. After the 37°C incubation, place the reactions on ice and proceed immediately to the reverse transcription.
2. Reverse Transcription to Synthesize First Strand cDNA:
  1. Prepare Reverse Transcription Master Mix in a nuclease-free tube at room temp in the order shown on the calculation sheet. Assemble enough master mix for all the samples in the experiment, including ≤5% overage to cover pipetting error. Gently vortex to make a homogenous mixture without inactivating the enzyme, then centrifuge for ~5 s to collect the master mix at the bottom of the tube.
  2. Transfer 10 µl of Reverse Transcription Master Mix to each sample, mix thoroughly by gentle vortexing, and follow with a quick spin to collect the reaction.
  3. Place the samples in a 42°C incubator. Incubate for 2 hr at 42°C, then centrifuge briefly (~5 s) to collect the reaction at the bottom of the tube.
  4. Place the tubes on ice and immediately proceed to the second strand cDNA synthesis.
3. Second Strand cDNA Synthesis:
  1. On ice, prepare a Second Strand Master Mix by mixing the following reagents in the order shown in the calculation sheet. Assemble enough master mix for all the samples in the experiment, including ≤5% overage to cover pipetting error. Gently vortex to make a homogenous mixture without inactivating the enzymes, then centrifuge for ~5 s to collect the master mix at the bottom of the tube.
  2. Transfer 80 µl of Second Strand Master Mix to each sample, mix thoroughly by gentle vortexing, and follow with a quick spin to collect the reaction.
  3. Place the samples in a 16°C thermal cycler. It is important to cool the thermal cycler block to 16°C before adding the reaction tubes because subjecting the reactions to temperatures >16°C will compromise aRNA yield.
  4. Incubate 2 hr at 16°C Incubate 2 hr in a 16°C thermal cycler. If the lid temperature cannot be adjusted to match the 16°C block temperature, cover the reactions with the heated lid turned off, or if the lid cannot be turned off—do not cover the tubes with it.
  5. Place reactions on ice briefly. Do not leave the reactions on ice for long periods of time.
4. 5.4) cDNA Purification:
  1. All centrifugations in this purification procedure should be done at 10,000 x g (typically ~10,000 rpm) at room temp. Check the cDNA Binding Buffer for precipitation before using it. If a precipitate is visible, redissolve it by warming the solution to 37°C for up to 10 min and vortexing vigorously. Cool to room temp before use.
  2. Before beginning the cDNA purification, preheat the 10 ml bottle of Nuclease-free Water to 50°C for at least 10 min.
  3. Add 250 µl of cDNA Binding Buffer to each sample and mix thoroughly by gently vortexing.
  4. Centrifuge for ~1 min at 10,000 g, or until the mixture is through the filter. Discard the flow-through and replace the cDNA Filter Cartridge in the wash tube.
  5. Apply 500 µl Wash Buffer to each cDNA Filter Cartridge. Centrifuge for ~1 min at 10,000 g, or until all the Wash Buffer is through the filter. Discard the flow-through and spin the cDNA Filter Cartridge for an additional minute to remove trace amounts of EtOH.
  6. Transfer cDNA Filter Cartridge to a cDNA Elution Tube. Elute cDNA with 20 µl of 50°C Nuclease-free Water. It is important to use Nuclease-free Water that is at 50°C for the cDNA elution. Colder water will be less efficient at eluting the cDNA, and hotter water (>55°C) may result in reduced aRNA yield.
  7. To the center of the filter in the cDNA Filter Cartridge, apply 20 µl of preheated (50°C) Nuclease-free Water. Leave at room temperature for 2 min and then centrifuge for ~1.5 min at 10,000 x g, or until all the Nuclease-free water is through the filter. The double-stranded cDNA will now be in the elute (~16 µl).
  8. The purified cDNA can be stored overnight at –80°C at this point if desired.
5. In Vitro Transcription to Synthesize aRNA
  1. It is recommended to use a hybridization oven because of their uniform temperature, and because it is extremely important that condensation does not form inside the tubes. We strongly recommend a 14 hr IVT reaction incubation to maximize aRNA yield. For the highest aRNA yield, conduct the IVT in a 40 µl final reaction volume.
  2. At room temp, assemble an IVT Master Mix by adding reagents in the order listed on the calculation sheet. Mix well by gently vortexing. Centrifuge briefly (~5 s) to collect the IVT Master Mix at the bottom of the tube and place on ice.
  3. Transfer IVT Master Mix to each sample following the guidelines of the calculation sheet, mix thoroughly by gentle vortexing, centrifuge briefly to collect the reaction, and place the tubes in a 37°C incubator.
  4. Incubate for 14 hr at 37°C.
  5. After the incubation, add Nuclease-free Water to each aRNA sample to bring the final volume to 100 µl. Mix thoroughly by gentle vortexing. Place the diluted aRNA on ice if the aRNA purification step will be done immediately. Alternatively, the aRNA can be stored at –20°C.
6. aRNA Purification
7. All centrifugations in this section should be done at 10.000 x g (typically ~10,000 rpm).
8. Before beginning the aRNA purification preheat the 10 ml bottle of Nuclease-free Water to 55°C for > 10 min.
9. Add 350 µl of aRNA Binding Buffer to each aRNA sample. Mix thoroughly by gentle vortexing, and proceed to the next step immediately.
10. Add 250 µl of ACS grade 100% EtOH to each aRNA sample, and mix by pipetting the mixture up and down 3 times. Do not vortex to mix. Proceed immediately to the next step as soon as you have mixed the EtOH into each sample.
11. Place an aRNA Filter Cartridge in an aRNA Collection Tube, and pipette each sample mixture onto the center of the filter in the aRNA Filter Cartridge.
12. Centrifuge for ~1 min at 10,000 x g. Continue until the mixture has passed through the filter. Discard the flow-through and replace the aRNA Filter Cartridge in the aRNA Collection Tube.
13. Apply 650 µl Wash Buffer to each aRNA Filter Cartridge. Centrifuge for ~1 min at 10,000 x g, or until all the wash solution is through the filter. Discard the flow-through and spin the aRNA Filter Cartridge for an additional ~1 min to remove trace amounts of EtOH.
14. Transfer Filter Cartridge(s) to a fresh aRNA Collection Tube. To the center of the filter, add 75 µl Nuclease-free Water that is preheated to 50–60°C. Replace the container of Nuclease-free Water in the 50–60°C incubator.
15. Leave at room temperature for 2 min and then centrifuge for ~1.5 min at 10,000 x g, or until the solution is through the filter.
16. Repeat the elution with a second 75 µl of Nuclease-free Water. The aRNA will now be in the aRNA Collection Tube in ~150 µl of Nuclease-free Water. Discard the aRNA Filter Cartridge.
17. Store aRNA at –80°C and minimize repeated freeze-thawing.

#### Part 7: cDNA synthesis

*This part uses the Promega Universal RiboClone cDNA Synthesis System kit with slight modification to the manufacturer’s instructions. Standard input 2 µg; for 454 sequencing, it is recommended to begin with 10 µg. If the concentration is too low, the aRNA can be concentrated with either EtOH precipitation or vacuum concentration. If 10 µg is used as RNA input then scale all the reagents up by a factor of 5The enzymes come in units different each batch, thus volumes have to be calculated each time.*

1. First Strand Synthesis:
  1. Take a 0.5 ml PCR tube and add:

**Table:** 

mRNA sample	2 μg (or 10 μg if used for 454 sequencing)
Random Hex Primer (0.5 mg/ml)	2 μl
Nuclease free water to volume	0 (or non is sample was dilute)
**Total Volume**	**15 μl**
  2. Heat the reaction to 70°C for 10 min. Chill tube on ice for 5 min and spin briefly to collect the solution at the bottom of the tube.
  3. Add the following components in the order shown:

**Table:** 

Sample	15 μl
First Strand 5X Buffer	5 μl
RNasin Ribonuclease Inhibitor 40 u	1 μl ... 40 u/μl
**Total Volume**	**21 μl**
  4. Mix reaction and spin briefly. Heat mixture at 37°C for 5 min.
  5. Add the following components:

**Table:** 

Sample from step 1	21.0 μl
Sodium Pyrophosphate, 40 mM	2.5 μl
AMV Reverse Trancriptase 30 u	1.5 μl ... 22 u/μl
Nuclease free water	0.0 μl
**Total Volume**	**25.0 μl**
  6. Incubate reaction for 1 h at 37°C in hybridization oven.
  7. After incubation place reaction on ice.
2. Second-Strand Synthesis:
  1. Add the following components:

**Table:** 

First-Strand reaction	25.0 μl
Second Strand 2.5X Buffer	40.0 μl
BSA, 1 mg/ml	5.0 μl
DNA Polymerase I 23 u	3.0 μl
RNase H 0.8 u	0.5 μl ... 1.5 u/μl
Nuclease free water	26.5 μl
**Total Volume**	**100.0 μl**
  2. Mix gently by flicking the tube. Incubate the reactions at 14°C for 2 h in a thermocycler. It is important to cool the thermocycler to 14°C before adding the reaction tubes. Do not let the reaction get above 14°C before or during the reaction.
  3. To terminate the reaction, add 2 units of T4 DNA Polymerase per μg input mRNA to the reaction. Incubate for 10 min at 14°C.
  4. Stop the reaction by adding 10 μl of 500 mM DEPC treated EDTA and place on ice.
  5. Clean the cDNA using either an EtOH precipitation or the Promega Wizard DNA-Cleanup System according to the manufacturer’s instructions. Store samples at -80°C. Double-stranded cDNA can be directly pyrosequenced at this point.

## Discussion

The investigation of gene expression by natural microbial communities has become common in recent years as a means to explore the ecological roles and functions of microorganisms. Used in combination with the detection, quantification, and characterization of marine microorganisms, analyses of gene expression can be used to link phylogeny to function in natural microbial communities. Many techniques for targeting functional gene expression rely on specific probes or primer sets designed for genes of known sequence. In contrast, environmental transcriptomics can be used to examine gene expression without constraints imposed by existing sequence data and with preference for those genes being actively expressed. Analysis of the mRNA pool in the environment can therefore provide one of the most effective ways of discovering connections between key activities and the organisms that mediate them.

The initial application of environmental transcriptomics provided one of the first views of the composition and dynamics of the bacterial mRNA pool in a natural ecosystem ^3^. Analysis of the expressed genes in transcript libraries from both a coastal salt marsh (Sapelo Island, GA) and an alkaline, hypersaline lake (Mono Lake CA) revealed gene sequences of biogeochemical interest, including environmental variants of several functional genes such as chitinases and sulfur oxidation genes that were specific to each of these ecosystems. It also provided evidence for novel, unexpected processes such as the microbial degradation of vascular plant- and algal-derived polyamines as a possible carbon and nitrogen sources.

As molecular techniques evolve to reduce the limitations associated with working with RNA from environmental samples and sequencing technologies improve, environmental transcriptomics has become a more widely used technique. It has been used to examine the functions of microorganisms in the surface ocean ^6^ and to compare day and night functional gene expression within the same environment ^7^. Environmental transcriptomics can also be used for gaining a better understanding of microbial responses to specific environmental factors and biogeochemistry. Genes or functions discovered using this technique can serve as targets for more quantitative studies of gene expression such as microarrays and quantitative PCR.
